# Hyperuricemia is accompanied by elevated peripheral CD4^+^ T cells

**DOI:** 10.1038/s41598-023-39775-2

**Published:** 2023-08-02

**Authors:** Qiuyang Yu, Zhengyi Sun, Ying Wang, Xue Du, Jing Huang, Liying Wang

**Affiliations:** 1 Department of Laboratory Medicine, The First Hospital of Jilin University, Jilin University, Changchun, 130021 Jilin People’s Republic of China; 2 Institute of Pediatrics, The First Hospital of Jilin University, Jilin University, Changchun, 130021 Jilin People’s Republic of China; 3 Department of Molecular Biology, College of Basic Medical Sciences, Jilin University, Changchun, 130021 Jilin People’s Republic of China

**Keywords:** Immunology, Lymphocytes, Endocrine system and metabolic diseases, Rheumatic diseases

## Abstract

Hyperuricemia (HUA) makes a chronic inflammation status, which affects immune cells. The association between HUA and immune cells, such as monocytes and neutrophils, has been extensively studied. However, studies on HUA and lymphocytes are still limited. We selected 1543 healthy participants and 258 individuals with HUA to analyze the correlation between serum uric acid (SUA) levels and immune cells, and 98 healthy participants and 16 individuals with HUA were used to study the relationship between SUA levels and cytokine levels. Then, we used soluble UA to stimulate peripheral blood mononuclear cells in vitro and examined lymphocyte subset counts and activation by flow cytometry. The results revealed that the number of lymphocytes in the HUA group was significantly increased, particularly CD4^+^ T cell numbers, which were higher than those in the total population (*P* = 0.0019), females (*P* = 0.0142), and males (*P* = 0.0199) of the healthy control group. Concomitantly, interleukin (IL)-4 and IL-10 levels significantly increased in people with HUA (*P* = 0.0254; *P* = 0.0019). In vitro, soluble UA promoted the proliferation and activation of CD4^+^ T and CD19^+^ B cells. Thus, HUA is accompanied by elevated peripheral CD4^+^ T cells and may cause a Th2-dominant immune status.

## Introduction

Owing to the improvements in living standards, hyperuricemia (HUA) has become a major public health problem and is currently the second most common metabolic disease after diabetes^1^. HUA is caused by soluble uric acid (UA) levels in the body exceeding the threshold. As soluble UA levels continue to rise, UA crystals form and deposit in renal tubules, joints, and soft tissues, causing organ and tissue damage and leading to various diseases, the most common of which is gout^2^. Indeed, gout has been observed in only 36% of patients with HUA. Most patients only exhibit elevated soluble UA levels without deposited UA crystals^3–5^. Therefore, HUA deserves more clinical attention.

Soluble UA that causes HUA is the end product of degradation of purine and purine-containing substances^6^. At physiological pH, UA in the body exists mainly in its soluble form^7^. In the early days of human evolution, soluble UA was broken down by uricase. However, with the loss of uricase, the kidney became the major site for soluble UA excretion; only 10% of soluble UA is excreted in the urine while the remaining 90% is reabsorbed^8^. This implies that HUA may be essential to the body.

Soluble UA has been found to drive epigenetic reprogramming in innate immune cells, causing a sustained increase in interleukin (IL)-1β gene transcription along with a persistent inflammatory response and increased responsiveness to secondary stimulation^9^. The recognition that soluble UA has such key proinflammatory roles in innate immunity raises vital questions of its importance in adaptive immunity. Previous studies have shown that soluble UA increases the expression of immune cell mediator CD40, which connects innate and adaptive immunity by activating dendritic cells, and enhances antigen presentation to T cells^10,11^. Moreover, soluble UA increased CD86 expression when co-cultured with primary bone marrow-derived dendritic cells, providing co-stimulatory signals for T cells to initiate the immune response^12^. Furthermore, depletion of soluble UA suppressed the immune response related to dying cells^13^. One study on HUA in male individuals described an inverse correlation between serum UA (SUA) levels and natural killer cell counts. Given these findings, we herein explore the relationship between soluble UA, which results in HUA, and lymphocytes (LY).

Hence, we retrospectively analyzed the association of SUA levels with LY and cytokines in healthy participants and individuals with HUA. In addition, we conducted in vitro experiments to further study the effect of soluble UA on LY. Our study not only provides clinical data for determining the relationship between HUA and LY but also provides valuable clues for future research on related mechanisms.

## Results

### Correlation of SUA levels with immune cells in human peripheral blood

**Figure 1:**
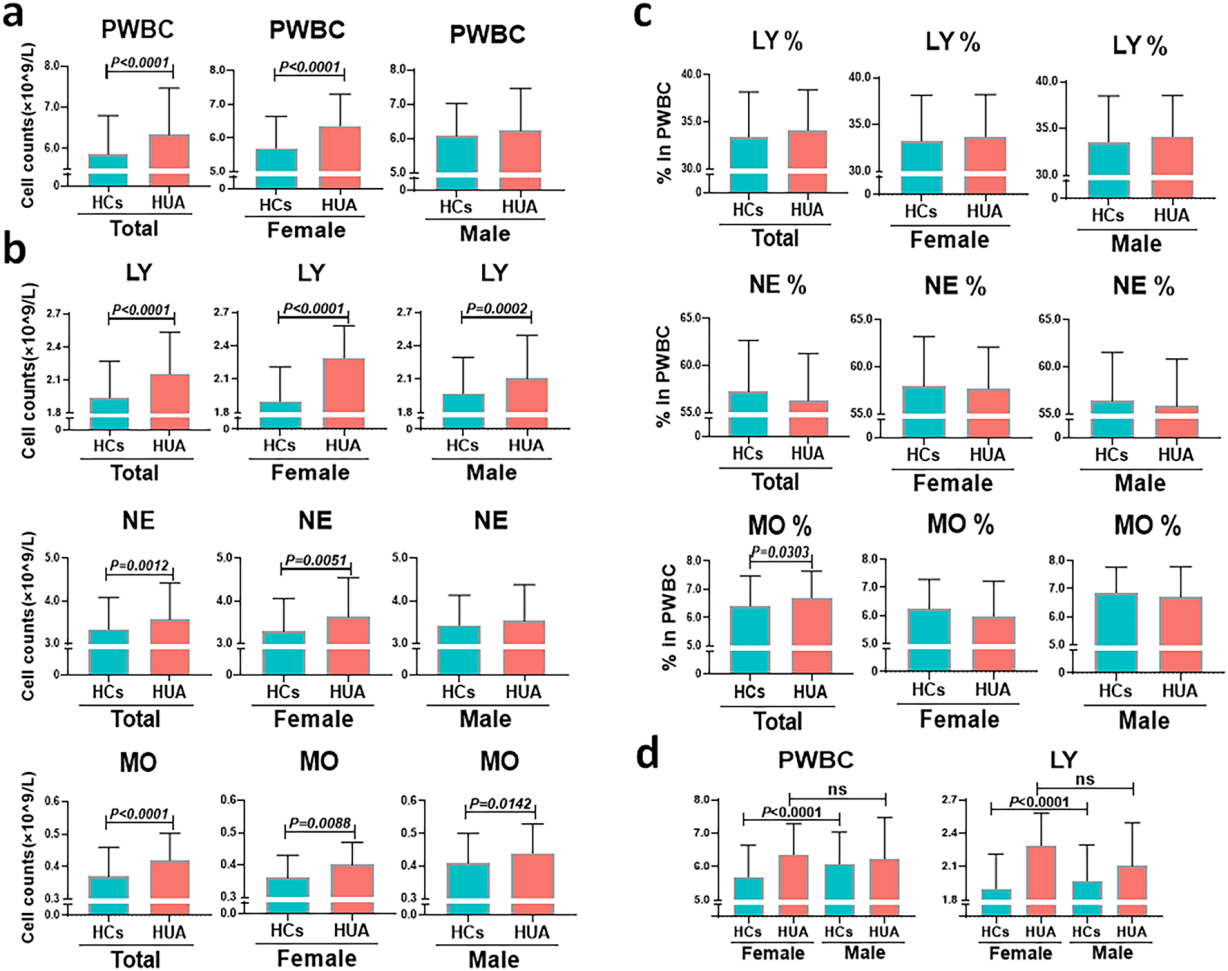
SUA-related changes in immune cells. (**a**) Comparison of the number of PWBC in HCs and HUA groups. (**b**) Comparison of the number of LY, NE, and MO in HCs and HUA groups. (**c**) Comparison of the percentages of LY, NE, and MO in HCs and HUA groups. **(d)** Comparison of the number of PWBC and LY in HCs and HUA groups with different sex. Cases of HCs group in the total population = 1543, cases of HUA group in the total population = 258, cases of HCs group in the female population = 979, cases of HUA group in the female population = 77, cases of HCs group in the male population = 564, cases of HUA group in the male population = 181. In the box-and-whisker plots, the centerline corresponds to the median, the box corresponds to the IQR, and the whiskers correspond to 1.5 × IQR, while outlier points are plotted individually where present. Statistical significance was indicated by *P* < 0.05. *P* values were calculated using the Mann‒Whitney U test. *HCs* healthy controls*; HUA* hyperuricemia*; PWBC* peripheral white blood cell; *LY* lymphocytes; *NE* neutrophils; *MO* monocyte; *IQR* interquartile range; *SUA* serum uric acid.

We compared the difference in the number of PWBC, LY, NE, and MO and the percentage of LY, NE, and MO between HCs and individuals with HUA. The results indicated that these two groups had similar counts of PWBC in the male population, but the counts of PWBC in the total and female population were significantly higher in the individuals with HUA than in the HCs (median 6.34 (5.40, 7.47) vs. 5.86 (5.00, 6.80) and 6.36 (5.64, 7.29) vs. 5.69 (4.87, 6.64), respectively; both *P* < 0.0001, Fig. 1a). In addition, in the total and female population with HUA, although the numbers of LY, NE, and MO were higher than those in HCs, the change in LY was the most obvious, with 11% (total population) and 21% (female population) higher increase in the individuals with HUA than in HCs (median 2.16 (1.79, 2.53) vs. 1.94 (1.62, 2.27) and 2.29 (1.80, 2.58) vs. 1.90 (1.60, 2.21), respectively; both *P* < 0.0001, Fig. 1b). LY counts were not significantly different between males and females with HUA; however, they tended to be elevated in females (Fig. 1d). No significant differences were observed in the percentages of LY and NE between the two groups, while the percentage of MO was elevated in individuals with HUA in the total population (median 6.41 (5.48, 7.48) vs. 6.70 (5.71, 7.64), *P* = 0.0303, Fig. 1c). As a result, individuals with HUA may have an increase in LY number, which is more pronounced in females.

### Increased peripheral blood CD4^+^ T cell number in individuals with HUA

**Figure 2:**
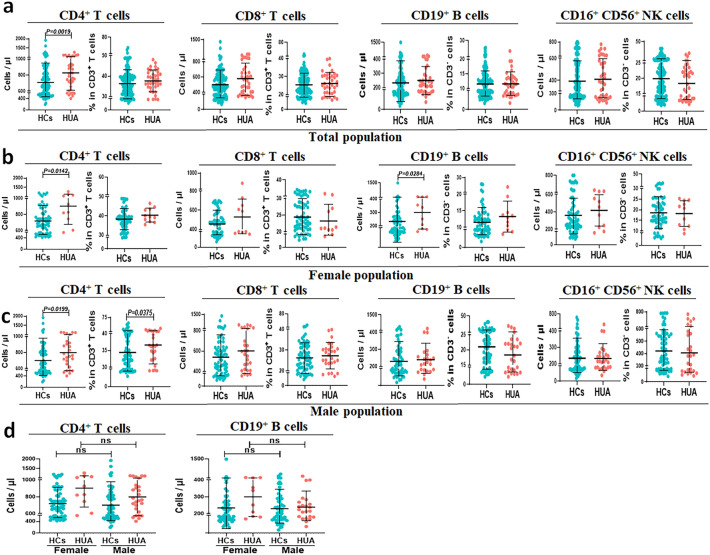
SUA-related changes in the counts of LY subsets. (**a**) Comparison of the number and percentage of LY subsets in HCs and HUA groups in the total population. (**b**) Comparison of the number and percentage of LY subsets in HCs and HUA groups in the female population. (**c**) Comparison of the number and percentage of LY subsets in HCs and HUA groups in the male population. “**(d)** Comparison of the number of CD4^+^ T cells and CD19^+^ B cells in HCs and HUA groups with different sex. Cases of HCs group in the total population = 98, cases of HUA group in the total population = 38, cases of HCs group in the female population = 32, cases of HUA group in the female population = 11, cases of HCs group in the male population = 66, cases of HUA group in the male population = 27. Each point in the scatter plot represents a person’s data, and the black line shows the mean value. Statistical significance was indicated by *P* < 0.05. *P* values were calculated using the Mann‒Whitney *U* test. *HCs* healthy controls*; HUA* hyperuricemia; *LY* lymphocyte; *SUA* serum uric acid.

To further explore which type of LY was mainly associated with HUA, we compared the numbers and percentages of CD4^+^ T, CD8^+^ T, CD19^+^ B, and CD16^+^CD56^+^ NK cells between the HCs and individuals with HUA. Compared with the HCs, CD4^+^ T cell counts were significantly increased in females and males with HUA (median 673.20 (549.10, 814.00) vs. 821.00 (639.70, 1024.00) in the total population, *P* = 0.0019; 694.40 (581.00, 812.90) vs. 877.00 (710.00, 1092.00) in the female population, *P* = 0.0142; 647.00 (534.00, 817.00) vs. 764.00 (621.00, 938.90) in the male population, *P* = 0.0199, Fig. 2a–c), and there was no difference in CD8^+^ T and CD16^+^CD56^+^ NK cells. Surprisingly, in females with HUA, the number of CD19^+^ B cells also increased significantly (median 199.00 (152.20, 268.00) vs. 295.00 (188.00, 430.10), *P* = 0.0284, Fig. 2b). Furthermore, both CD4^+^ T and CD19^+^ B cell counts in females with HUA tended to be higher than those in males (Fig. 2d). Hence, HUA may mainly be associated with an increase in CD4^+^ T cells.

### Increased IL-4 and IL-10 levels in individuals with HUA

**Figure 3:**
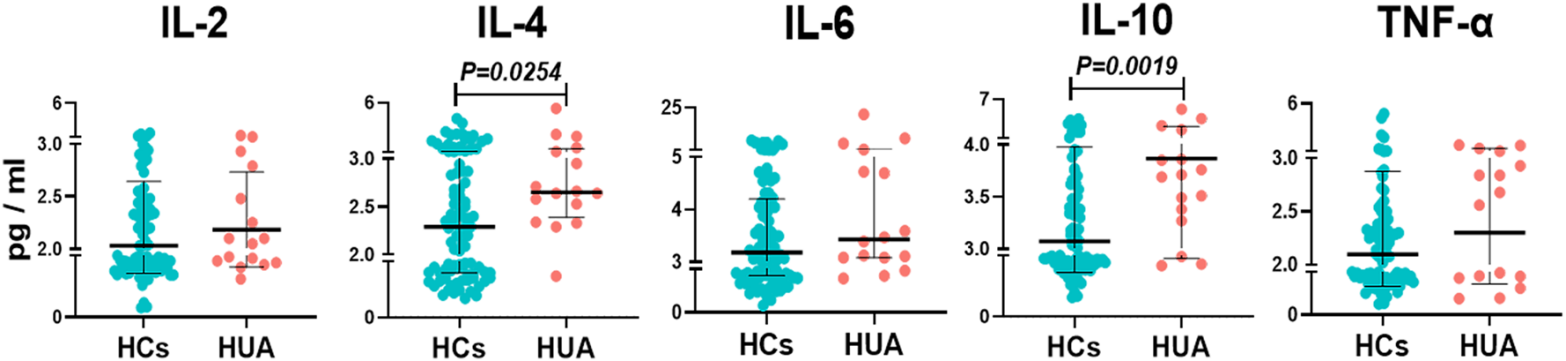
SUA-related changes in cytokine levels. Cases in the HCs group = 92, cases in the HUA group = 16. Each point in the scatter plot represents a person’s data. The black line shows the mean value in IL-2, Il-10, and TNF-α. The black line shows the median values of IL-4 and IL-6. Statistical significance was indicated by *P* < 0.05. *P* values were calculated using the paired t test for IL-2, IL-10, and TNF-α. *P* values were calculated using the Mann‒Whitney U test for IL-4 and IL-6. *HCs* healthy controls*; HUA* hyperuricemia; *IL* interleukin; *SUA* serum uric acid; *TNF- α* tumor necrosis factor-alpha.

Cytokines are closely associated with the functions of LY. Therefore, we analyzed the changes in IL-2, IL-4, IL-6, IL-10, and TNF-α levels between HCs and individuals with HUA. The levels of IL-4 and IL-10 were higher in individuals with HUA (median 2.29 (1.60, 3.05) vs. 2.65 (2.39, 3.21) and mean 3.07 ± 0.90 vs. 3.87 ± 1.01, respectively; *P* = 0.0254 and *P* = 0.0019), and no significant changes were found in the levels of IL-2, IL-6, and TNF-α (Fig. 3).

### Effect of soluble UA on LY count in vitro

**Figure 4:**
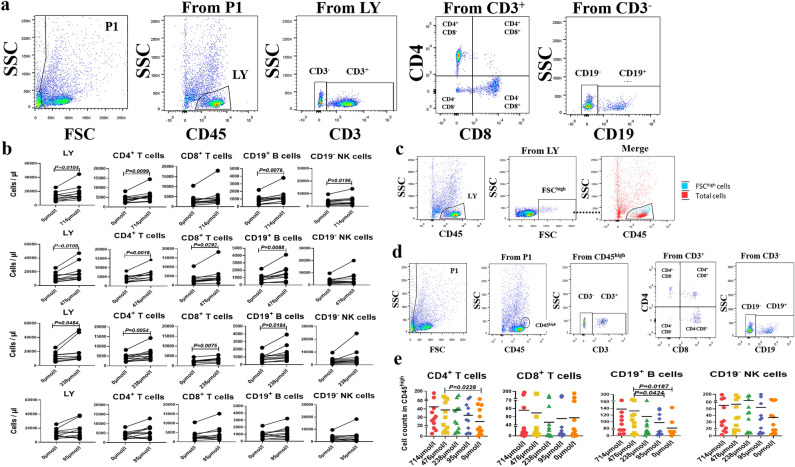
Soluble UA promotes LY proliferation in vitro. (**a**) The gating strategy for analyzing stimulated and unstimulated LY in vitro. (**b**) Changes in LY and its subsets treated with 714, 476, 238, and 95 µmol/L soluble UA relative to untreated cells (n = 11). (**c**) Expression of CD45 in FSC^high^ LY cells. (**d**) The gating strategy for analyzing stimulated CD45^high^ cells in vitro. (**e**) Comparison of the counts of CD4^+^ T cells, CD8^+^ T cells, CD19^+^ B cells, and CD19^−^ NK cells in CD45^high^ LY cells with or without stimulation by soluble UA. Each point in the scatter plot represents a person’s data, and the black line shows the mean value Statistical significance was indicated by *P* < 0.05. *P* values were calculated using the paired t test. *LY* lymphocyte; *NK cells* natural killer cells; *UA* uric acid.

To determine whether soluble UA affects the LY count, we stimulated LY with different concentrations of soluble UA. Compared with the soluble UA concentration of 0 µmol/L, the total number of LY in the medium with soluble UA concentrations of 714, 476, and 238 µmol/L was significantly increased (mean 12,263 ± 6232 vs. 17,519 ± 10,352, 12,263 ± 6232 vs. 18,936 ± 12,379, and 12,263 ± 6232 vs. 19,947 ± 15,592, respectively; *P* = 0.0104, *P* = 0.0108, and *P* = 0.0484). Soluble UA concentration of 714 µmol/L was associated with an increase in the counts of CD4^+^ T (mean 4038 ± 1837 vs. 5934 ± 3132, *P* = 0.0099), CD19^+^ B (mean 871 ± 552 vs. 1330 ± 894, *P* = 0.0076), and CD19^−^ NK cells (mean 3297 ± 2441 vs. 4424 ± 3639, *P* = 0.0196), and soluble UA concentrations of 476 and 238 µmol/L were associated with an increase in the counts of CD4^+^ T (mean 4038 ± 1837 vs. 6200 ± 3153 and 4038 ± 1837 vs. 5986 ± 3177, respectively; *P* = 0.0018 and *P* = 0.0054), CD8^+^ T (mean 3238 ± 2605 vs. 4880 ± 4598 and 3238 ± 2605 vs. 3524 ± 3167, respectively; *P* = 0.0292 and *P* = 0.0075), and CD19^+^ B cells (mean 871 ± 552 vs. 1473 ± 1068 and 871 ± 552 vs. 1368 ± 1036, respectively; *P* = 0.0088 and *P* = 0.0184, Fig. 4b). However, the difference did not reach statistical significance in LY treated with soluble UA at 95 µmol/L compared with that at 0 µmol/L. These results confirm that soluble UA has the ability to cause an increase in LY counts.

Next, to explore whether soluble UA can activate LY, we analyzed the changes in LY size and CD45 expression under soluble UA stimulation. Intriguingly, we observed a cluster of FSC^high^ cells in LY stimulated by soluble UA, and this cluster was concentrated in the CD45^high^ region (Fig. 4c). Subsequently, we compared the number of CD4^+^ T, CD8^+^ T, CD19^+^ B, and CD19^−^ NK cells in CD45^high^FSC^high^ cells with or without soluble UA stimulation (Fig. 4d). Under stimulation with 476 µmol/L soluble UA, the counts of CD4^+^ T and CD19^+^ B cells among the CD45^high^FSC^high^ cells significantly increased (mean 38.91 ± 31.58 vs. 25.00 ± 19.20 and 132.70 ± 138.70 vs. 80.45 ± 95.39, respectively; *P* = 0.0228 and *P* = 0.0187, Fig. 4e). The findings indicate the association of soluble UA with CD4^+^ T and CD19^+^ B cell activation.

### Relationship between SUA levels and LY counts in different diseases

**Figure 5:**
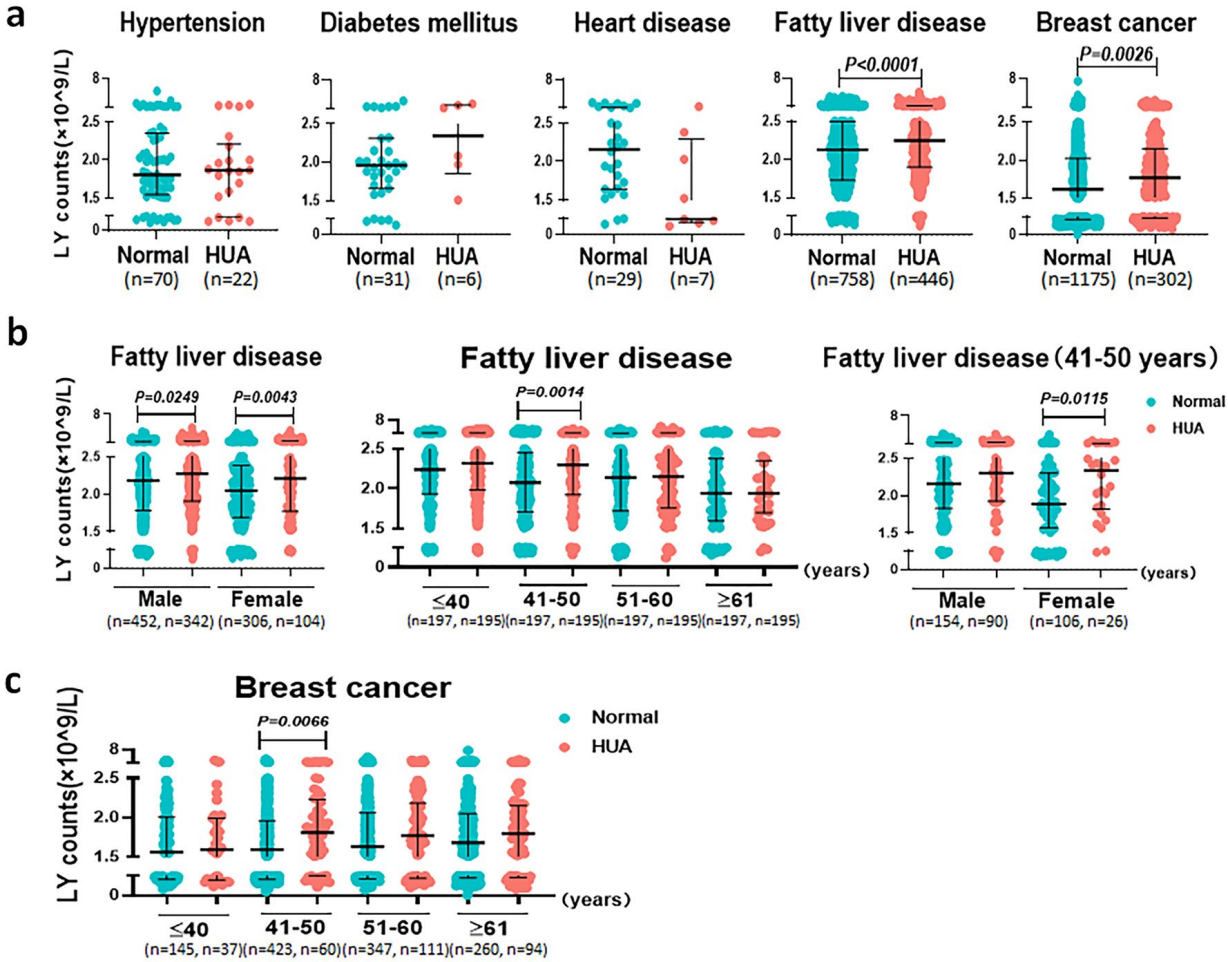
The difference of LY counts in different diseases with and without HUA. (**a**) Comparison of the number of LY between the normal SUA group and HUA group in different diseases. (**b**) Comparison of the number of LY between normal SUA group and HUA group in fatty liver disease patients of different sex and age. (**c**) Comparison of the number of LY between normal SUA group and HUA group in breast cancer patients of different age. Each point in the scatter plot represents a person’s data, and the black line shows the mean value. Statistical significance was indicated by *P* < 0.05. *P* values were calculated using the Mann‒Whitney *U* test. *HCs* healthy controls*; HUA* hyperuricemia; *LY* lymphocyte.

To explore whether different diseases combination with HUA influences the number of LY, we compared the number of LY in hypertension, heart disease, diabetes, fatty liver disease, and breast cancer with and without HUA. Our findings revealed significantly higher LY counts in patients with fatty liver disease and breast cancer combined with HUA (median 2.13 (1.73, 2.50) vs. 2.25 (1.90, 2.65) and 1.62 (1.25, 2.02) vs. 1.77(1.38, 2.15), respectively; *P* < 0.0001 and *P* = 0.0026, Fig. 5a). No such difference was observed in patients with hypertension, diabetes mellitus, and heart disease combined with HUA. Furthermore, LY counts were increased in both male and female patients with fatty liver disease combined with HUA (median 2.17 (1.73, 2.50) vs. 2.25 (1.90, 2.65), *P* = 0.0249 and median 1.62 (1.25, 2.02) vs. 1.77 (1.38, 2.15), *P* = 0.0043, respectively). In female patients with fatty liver disease along with HUA aged 41–50 years, LY counts were substantially higher (median 1.64 (1.73, 2.50) vs. 1.84 (1.90, 2.65), *P* = 0.0115, Fig. 5b). Similarly, LY counts also increased in female patients with breast cancer combined with HUA aged 41–50 years (median 1.59 (1.23, 1.96) vs. 1.81 (1.49, 2.23), *P* = 0.0066, Fig. 5c). Overall, fatty liver disease and breast cancer combined with HUA may affect the number of LY.

## Discussion

The growing recognition that soluble UA plays a complex role in immune biology has stimulated strong interest in its relationship with immune cells. However, there are few existing analyses of the relationship between soluble UA and LY and even fewer domestic studies; therefore, our study is the first to combine retrospective analyses and in vitro experiments to investigate the effect of soluble UA on LY. We found that elevated soluble UA was mainly accompanied by an increase in CD4^+^ T cell counts and IL-4 and IL-10 levels, implying that HUA may lead to a Th2-dominated immune status.

Studies in patients with HUA have shown that an increase in SUA concentration is related to inflammatory markers such as PWBC, C reactive protein, and circulating innate immune cytokines such as IL-6, IL-1Ra, TNF, or IL-18^15,16^. This is in line with our clinical results. CD4^+^ T cells are divided into Th1 and Th2 cells, of which the latter secrete IL-4, IL-6, and IL-10 to assist humoral immunity^17^. Therefore, we speculated that the cytokine environment of the HUA population might shift to Th2 cytokine to regulate humoral immunity. Since humoral immunity is mainly mediated by B cells, this is consistent with our in vitro experimental results and further confirms our hypothesis that individuals with HUA may be accompanied by enhanced humoral immunity. However, we need to analyze the relationship between soluble UA and levels of complement and antibodies to further explain our assumption.

Studies have shown that enhanced Th2-dominated immune response is related to allergic and autoimmune diseases mainly due to the fact that Th2 immune response can promote humoral immunity^18,19^. This infers that individuals with HUA are susceptible to allergic and autoimmune diseases. Additionally, it has been reported that the X chromosome harbors genes that can control immune function. Given that females have two X chromosomes, females have stronger immunity and higher levels of physiological antibodies than males^20^. However, excessive autoantibodies can cause tissue damage, making females more vulnerable to autoimmune diseases. Previous studies showed that females had lower SUA levels compared to males because urate precipitation can be induced by male hormones while it is resisted by female estrogen^21,22^. As a result, females may be more sensitive to increased SUA than males. Therefore, females with HUA may develop stronger Th2 immune response than males, accumulating more antibodies with an increased risk of autoimmune disease. This may explain why the number of CD4^+^ T and CD19^+^ B cells in females tended to be higher than those in males in our clinical results. Moreover, we found a more significant increase in LY in females aged 41–50 years with fatty liver and breast cancer combined with HUA. This could be because females aged 41–50 years were mostly at menopausal status and had low levels of estrogen, leading to higher levels of SUA, which triggered a stronger immune response.

Clinical and epidemiological studies have shown that HUA increases the risk of developing chronic inflammatory disorders, including hypertension, acute and chronic kidney disease, obesity, metabolic syndrome, fatty liver, and diabetes mellitus^23,24^. LY counts were increased when HUA was present with fatty liver disease and breast cancer, implying that elevated SUA levels may drive inflammation by promoting LY proliferation. However, we observed no link between SUA and LY in heart disease, hypertension, and diabetes, probably due to the small sample size. In addition, this was a comparative study of immune-related indicators between asymptomatic individuals with HUA and healthy individuals and did not consider whether there were differences between asymptomatic individuals with HUA and those with gout. Therefore, in the future, we need to expand the sample size and the type of study participants to perform in-depth research. Soluble UA can inhibit IL-1 receptor antagonist promoting TLR-induced proinflammatory cytokine IL-1β production by human primary cells^25^. IL-1β is a key mediator of inflammatory response, which not only induces the proliferation and aggregation of neutrophils but also activates LY. Although IL-1β is critical for host response and resistance to pathogens, it also exacerbates damage during chronic disease and acute tissue injury and is associated with infection susceptibility, immune tolerance, and vaccine biology^26^. In view of this, soluble UA may promote an increase in the LY population through the IL-1β signaling pathway, which may contribute to host defense but may also lead to aseptic inflammation and pathological consequences. Research found that soluble UA could enhance the anti-tumor response of Th2 cells, promote antibody production by B cells, and relate to CD8^+^ T and NK cells^27^. Moreover, studies have confirmed that soluble UA as an adjuvant can play a role in the anti-tumor immunity induced by dendritic cell vaccines^28,29^. It is suggested that SUA may play an anticancer role by regulating LY when breast cancer was complicated with HUA. In the future, we plan to conduct further studies to explore the mechanism by which soluble UA promotes LY proliferation and its potential effects on the body.

In conclusion, individuals with HUA exhibited increased CD4^+^ T cell counts and IL-4 and IL-10 levels, suggesting that high levels of soluble UA may enhance the Th2-dominated immune response. Thus, physicians should assess the immune status of patients with HUA and other diseases combined with HUA by detecting LY subgroup or Th1/Th2 cytokines, in order to guide clinical treatment and rational regulation of SUA levels. Besides, soluble UA can be used as an immune adjuvant in the management of certain disease treatments, revealing a new perspective for clinical therapeutics.

## Materials and methods

### Patients and general information

We randomly selected 1915 healthy participants physically examined at the First Hospital of Jilin University from July 2020 to September 2020 to conduct this retrospective study. HUA is defined by SUA level of two fasting tests on different days, under a normal purine diet: males > 7 mg/dL and females > 6 mg/dL^14^. Of the 1915 healthy participants, 274 individuals met the diagnostic criteria of HUA. All individuals included in this study did not have cancer, heart disease, diabetes mellitus, fatty liver, hypertension, gouty arthritis, or other inflammatory disease during the study period. All individuals with dysglycemia and dyslipidemia and taking antihypertensive drugs, urate-lowering drugs, diuretics, and immunosuppressants were excluded from the study. The 274 participants who were diagnosed with HUA did not have gout symptoms or gout. The general characteristics of the 1641 healthy controls (HCs) and 274 individuals with HUA included in our study are presented in Table 1. In this study, 1543 cases of HCs and 258 cases of HUA were included in the analysis of the correlation between SUA levels and immune cells, and 98 cases of HCs and 16 cases of HUA were included in the analysis of the correlation between SUA and cytokine levels. At the same time, we aimed to explore whether SUA levels affect the number of LY in different diseases, as well as the potential mechanism by which HUA causes other diseases. We randomly selected 92 patients with hypertension, 37 patients with diabetes, 36 patients with heart disease, 1204 patients with fatty liver disease, and 1477 patients with breast cancer to analyze the relationship between SUA levels and peripheral blood LY with and without HUA. None of them had any other comorbidities, and they were not on diuretics, aspirin, levodopa, immunosuppressants, or antitumor drugs. Table 2 presents the general information of the patients with these diseases. This study was conducted in accordance with the guidelines of the Declaration of Helsinki and approved by the Ethics Committee of the First Hospital of Jilin University (Approval No. 2021-678). This study was the retrospective study and the in vitro experimental study using peripheral blood lymphocytes. The in vitro experiments involve only the remaining blood samples prepared to be discarded after the use of routine laboratory tests, do not involve direct contact with the subject, and the results of the study are not used for the diagnosis of the subject. Based on this, the Ethics Committee of the First Hospital of Jilin University approved the waiver of informed consent and agreed to carry out retrospective study and in vitro experimental study. The waste samples after the in vitro study were properly disposed of according to the biological sample waste treatment method.

**Table 1:** Clinical characteristics of healthy participants and individuals with HUA.

Parameter (units)	Immune cells		Cytokines	
	HCs	HUA	HCs	HUA
Number of samples, n	1543	258	98	16
Females, n (%)	979 (63.5%)	77 (29.8%)	38 (38.8%)	6 (37.5%)
Age, years	43.49 ± 11.47	41.99 ± 12.60	50.69 ± 12.24	53.00 ± 8.38
SBP, mmHg	115.22 ± 12.07	118.63 ± 13.49	–	–
DBP, mmHg	70.80 ± 8.89	73.39 ± 8.34	–	–
AST, U/L	19.46 ± 8.97	21.17 ± 7.00	20.15 ± 5.09	24.64 ± 8.75
ALT, U/L	17.55 ± 16.05	22.93 ± 14.70	18.31 ± 8.17	24.51 ± 9.63
γ-GGT,U/L	19.54 ± 25.11	29.52 ± 24.70	21.21 ± 13.46	43.45 ± 34.05
ALP, U/L	56.66 ± 15.99	61.11 ± 19.08	67.50 ± 19.03	69.21 ± 14.23
TG, mmol/L	1.26 ± 0.71	1.84 ± 1.35	1.06 ± 0.26	1.22 ± 0.36
TC, mmol/L	4.98 ± 0.96	5.12 ± 1.04	4.48 ± 0.58	4.39 ± 0.46
HDL-C, mmol/L	1.30 ± 0.28	1.21 ± 0.25	1.28 ± 0.30	1.25 ± 0.34
LDL-C, mmol/L	2.87 ± 0.75	3.08 ± 0.82	2.59 ± 0.35	2.53 ± 0.55
UA, µmol/L	296.79 ± 1.57	464.29 ± 4.03	315.05 ± 6.76	447.44 ± 12.38
Cre, µmol/L	61.95 ± 13.31	72.98 ± 13.99	71.16 ± 14.51	73.98 ± 17.87
Glu, mg/dL	–	–	5.19 ± 0.43	5.12 ± 0.77

The measurement data are shown as mean ± SD; category variables are expressed as frequency/percentage. *SBP* systolic blood pressure; *DBP* diastolic blood pressure; *AST* aspartate transaminase; *ALT* alanine aminotransferase; γ-*GGT* γ-glutamyl transpeptidase; *ALP* alkaline phosphatase; *TG* triglycerides; *TC* total cholesterol; *HDL-C* HDL-cholesterol; *LDL-C* LDL-cholesterol; *Cre* creatinine; *Glu* glucose; *UA* uric acid; *HCs* healthy controls; *HUA* hyperuricemia.

**Table 2:** Clinical characteristics of patients with these diseases.

Parameter (units)	Hypertension		Diabetes mellitus		Heart disease		Fatty liver		Breast cancer	
	Without HUA	With HUA	Without HUA	With HUA	Without HUA	With HUA	Without HUA	With HUA	Without HUA	With HUA
Number of samples, n	70	22	31	6	29	7	758	446	1175	302
Females, n (%)	24 (34.3%)	2 (9.1%)	5 (16.1%)	2 (33.3%)	9 (31.0%)	1 (14.3%)	306 (40.4%)	104 (23.3%)	1175 (100%)	302 (100%)
Age, years	60.64 ± 11.69	61.95 ± 11.46	58.39 ± 11.53	62.33 ± 14.46	60.07 ± 11.58	57.57 ± 17.21	47.89 ± 10.80	43.84 ± 12.18	51.73 ± 10.19	54.52 ± 10.99
SBP, mmHg	149.80 ± 18.29	149.80 ± 17.53	124.70 ± 9.92	126.00 ± 10.14	117.80 ± 10.71	118.30 ± 15.39	121.00 ± 10.87	121.70 ± 9.99	–	–
DBP, mmHg	88.03 ± 11.25	86.86 ± 9.06	75.74 ± 6.36	70.00 ± 9.21	72.28 ± 7.82	68.86 ± 7.49	74.21 ± 8.23	75.83 ± 8.05	–	–
PWB, 10^9^/L	6.31 ± 1.78	6.51 ± 1.31	7.09 ± 2.04	8.32 ± 1.72	6.77 ± 2.04	6.86 ± 1.30	6.61 ± 1.56	6.92 ± 1.66	5.55 ± 2.27	5.78 ± 2.17
NE, 10^9^/L	3.72 ± 1.37	3.94 ± 0.93	4.41 ± 1.67	5.24 ± 1.03	3.92 ± 1.36	4.55 ± 1.43	3.84 ± 1.21	3.96 ± 1.31	3.38 ± 1.98	3.48 ± 1.86
MO, 10^9^/L	0.43 ± 0.14	0.48 ± 0.14	0.47 ± 0.16	0.43 ± 0.05	0.47 ± 0.15	0.48 ± 0.11	0.45 ± 0.14	0.47 ± 0.14	0.37 ± 0.18	0.39 ± 0.18
AST, U/L	22.26 ± 7.13	22.90 ± 6.98	19.90 ± 5.27	22.82 ± 10.19	21.58 ± 5.28	21.87 ± 6.60	22.05 ± 10.38	25.57 ± 12.12	28.62 ± 19.18	51.30 ± 161.8
ALT, U/L	20.90 ± 10.39	21.95 ± 14.10	19.66 ± 9.79	18.92 ± 11.19	19.10 ± 8.39	19.79 ± 7.39	25.21 ± 17.41	34.44 ± 24.89	26.83 ± 21.21	38.55 ± 59.51
γ-GGT, U/L	28.11 ± 23.08	48.93 ± 47.48	29.71 ± 27.32	33.24 ± 26.95	30.13 ± 23.97	20.97 ± 15.19	30.92 ± 28.73	43.80 ± 42.46	43.13 ± 68.91	77.14 ± 185.6
ALP, U/L	62.74 ± 15.71	74.52 ± 20.90	66.66 ± 20.92	72.42 ± 25.22	67.74 ± 16.66	63.39 ± 11.13	62.88 ± 16.13	63.71 ± 16.31	96.81 ± 54.93	120.6 ± 151.2
TG, mmol/L	1.91 ± 1.96	2.14 ± 1.39	1.16 ± 0.32	2.28 ± 1.38	1.55 ± 0.95	1.13 ± 0.40	2.06 ± 1.46	2.55 ± 1.77	1.82 ± 1.16	2.56 ± 1.72
TC, mmol/L	5.14 ± 1.27	5.13 ± 0.89	4.98 ± 1.25	5.02 ± 1.11	5.60 ± 1.13	4.72 ± 1.72	5.20 ± 1.07	5.41 ± 0.97	5.02 ± 1.00	5.34 ± 1.12
HDL-C, mmol/L	1.23 ± 0.31	1.12 ± 0.17	1.16 ± 0.32	1.15 ± 0.21	1.26 ± 0.24	1.23 ± 0.42	1.19 ± 0.26	1.17 ± 0.23	1.41 ± 0.34	1.29 ± 0.31
LDL-C, mmol/L	2.99 ± 0.89	3.148 ± 0.80	2.90 ± 0.98	2.92 ± 0.81	3.41 ± 0.84	2.77 ± 1.03	3.13 ± 0.81	3.34 ± 0.77	2.86 ± 0.74	3.09 ± 0.80
Cre, µmol/L	83.62 ± 95.40	91.78 ± 26.66	66.61 ± 13.22	95.48 ± 50.34	70.29 ± 13.99	106.7 ± 52.42	66.09 ± 13.07	73.91 ± 14.49	55.85 ± 8.81	64.16 ± 19.21
Glu, mg/dL	5.62 ± 1.30	5.96 ± 1.11	9.57 ± 2.53	9.74 ± 4.08	5.20 ± 0.64	5.14 ± 0.56	5.40 ± 1.06	5.37 ± 0.84	5.62 ± 1.41	5.84 ± 1.23

The measurement data are shown as mean ± SD. Categorical variables are expressed as frequency/percentage. *SBP* systolic blood pressure; *DBP* diastolic blood pressure; *PWBC* peripheral white blood cell; *NE* neutrophils; *MO* monocytes; *AST* aspartate transaminase; *ALT* alanine aminotransferase; *γ-GGT* γ-glutamyl transpeptidase; *ALP* alkaline phosphatase; *TG* triglycerides; *TC* total cholesterol; *HDL-C* HDL-cholesterol*; LDL-C* LDL-cholesterol; *Cre* creatinine; *Glu* glucose; *HUA* hyperuricemia.

### Detection of SUA concentration

Fasting venous blood was collected once in the morning. After separating the serum, the SUA concentration of all individuals was detected via the uricase-peroxidase method using a commercially available kit (Beckman Coulter, Inc. Brea, USA) and measured using a Beckman AU5841 automatic chemistry analyzer (Beckman Coulter, Inc.).

### Detection of immune cells in peripheral blood

Whole blood was used to detect the number and percentage of immune cells, including peripheral white blood cells (PWBC), LY, neutrophils (NE), and monocytes (MO). Counts and percentages were analyzed using a Sysmex XN-9000 hematology analyzer (Sysmex, Kobe, Japan). The NE-LY ratio was determined by dividing the NE count by the LY count.

### Detection of LY subsets and cytokines

**Figure 6:**
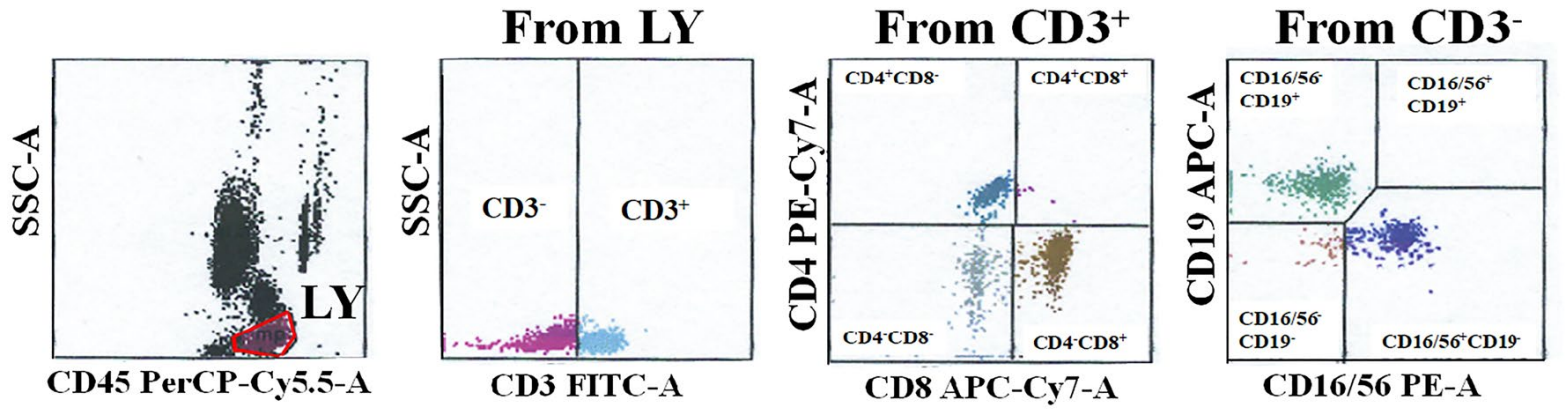
The gating strategy for analyzing the lymphocyte subsets. *LY* lymphocytes.

Peripheral blood LY subsets of HUA patients (n = 38) and healthy people (n = 98) were tested using a FACS Canto II flow cytometer (BD Biosciences, Franklin Lakes, NJ, USA) with a BD Multitest 6-colour TBNK agent, including CD45-PerCP-Cy5.5, CD3-FITC, CD4-PE-Cy7, CD8-APC-Cy7, CD19-APC, and CD16/CD56-PE (BD Biosciences, USA). The fluorescent beads in the absolute counting tube were used as an internal standard for the absolute counts of LY subsets. The absolute cell counts and percentages of LY subsets were analyzed using BD FACS Canto software (BD Biosciences, USA). The gating strategy used to analyze the LY subsets is shown in Fig. 6. The serum levels of cytokines (including tumor necrosis factor (TNF)-α, IL-2, IL-4, IL-6, and IL-10) were measured by BD FACS Canto II flow cytometry (BD Biosciences, USA) using a Cytometric Bead Array with a Human Th1/Th2 subgroup detection kit (Jiangxi Nuode Company, China) following the manufacturer’s instructions. Cytokine data were obtained using the BD Diva software (BD Biosciences, USA). The flow cytometry was conducted daily using standard and CS&T application settings to verify the comparability of the machine performance and results.

### Soluble UA preparation

The RPMI 1640 medium was prewarmed (37 °C), UA (U2875, Sigma; 5g) was added, and the medium was heated to 57 °C. The final concentration of soluble UA medium was 16 mg/dL. The medium was sterilized using 0.20 μm filters. Crystals were not detectable under these conditions (as confirmed using polarizing microscopy) nor did they develop during cell incubation.

### In vitro experiments

In the in vitro experiments, we used Ficoll density gradient centrifugation (Ficoll-Histopaque-1077; Sigma) to separate and purify human heparinized peripheral blood and to isolate peripheral blood mononuclear cells (PBMCs). We aspirated 1000 µL of whole peripheral blood and diluted it with 1000 µL of phosphate-buffered saline. The diluted liquid was slowly dropped onto the surface of a 1500 μL Ficoll separator and centrifuged at 450×*g* for 15 min (the speed of centrifugal ascent and descent was set to 1). After washing once with phosphate-buffered saline, the PBMCs (2 × 10^4^ per well in a 96-well plate) were cultured in RPMI 1640 medium supplemented with 10% fetal bovine serum and stimulated with 714, 476, 238, or 95 µmol/L soluble UA at 37 °C with 5% CO_2_. After 18 h of culture, the PBMCs in each well were collected. The expression of CD45, CD3, CD4, CD8, and CD19 in the PWBCs was detected via surface staining with PerCP-labelled anti-CD45, FITC-labelled anti-CD3, APC-Cy7-labelled anti-CD4, PE-Cy7-labelled anti-CD8, and Apc-labelled anti-CD19 monoclonal antibodies for 15 min, followed by flow cytometry measurements. LY was analyzed according to the gating strategy shown in Fig. 4a. We removed dead cells through the P1 gate, and then selected the cells with high CD45 expression as LY. The results were analyzed by FlowJo software (version 10.0.7r2).

### Statistical analysis

The Kolmogorov–Smirnov test (for number of cases > 50) or Shapiro–Wilk test (for number of cases < 50) was used to assess data distribution. When the data conformed to normal distribution and were described as means ± standard deviation, differences between two groups were compared using an independent samples *t*-test or paired *t*-test. Differences between multiple groups were compared using one-way analysis of variance. When the data did not show normal distribution and were described as medians ± interquartile range, differences were compared using the Mann–Whitney *U* test. In this case, differences between multiple groups were compared using the Kruskal–Wallis test. Differences with *P*-values < 0.05 were considered significant. Data were analyzed using SPSS version 20 (SPSS Inc., Chicago, IL, USA) and GraphPad Prism 8 (GraphPad Software Inc., San Diego, CA, USA).

### Ethics approval and consent to participate

This study was conducted in accordance with the guidelines of the Declaration of Helsinki and approved by the Ethics Committee of the First Hospital of Jilin University (Approval No. 2021-678). This study was the retrospective study and the in vitro experimental study using peripheral blood lymphocytes. The in vitro experiments involve only the remaining blood samples prepared to be discarded after the use of routine laboratory tests, do not involve direct contact with the subject, and the results of the study are not used for the diagnosis of the subject. Based on this, the Ethics Committee of the First Hospital of Jilin University approved the waiver of informed consent and agreed to carry out retrospective study and in vitro experimental study. The waste samples after the in vitro study were properly disposed of according to the biological sample waste treatment method.

## Data Availability

The datasets generated or analyzed during the current study are available from the corresponding author upon reasonable request.
